# Myoinositol Attenuates the Cell Loss and Biochemical Changes Induced by Kainic Acid Status Epilepticus

**DOI:** 10.1155/2016/2794096

**Published:** 2016-08-23

**Authors:** Lia Tsverava, Tamar Lordkipanidze, Eka Lepsveridze, Maia Nozadze, Marina Kikvidze, Revaz Solomonia

**Affiliations:** ^1^Institute of Chemical Biology, Ilia State University, 3/5 K. Cholokashvili Avenue, 0162 Tbilisi, Georgia; ^2^I. Beritashvili Center of Experimental Biomedicine, 14 L. Gotua Street, 0160 Tbilisi, Georgia

## Abstract

Identification of compounds preventing or modifying the biochemical changes that underlie the epileptogenesis process and understanding the mechanism of their action are of great importance. We have previously shown that myoinositol (MI) daily treatment for 28 days prevents certain biochemical changes that are triggered by kainic acid (KA) induced status epilepticus (SE). However in these studies we have not detected any effects of MI on the first day after SE. In the present study we broadened our research and focused on other molecular and morphological changes at the early stages of SE induced by KA and effects of MI treatment on these changes. The increase in the amount of voltage-dependent anionic channel-1 (VDAC-1), cofilin, and caspase-3 activity was observed in the hippocampus of KA treated rats. Administration of MI 4 hours later after KA treatment abolishes these changes, whereas diazepam treatment by the same time schedule has no significant influence. The number of neuronal cells in CA1 and CA3 subfields of hippocampus is decreased after KA induced SE and MI posttreatment significantly attenuates this reduction. No significant changes are observed in the neocortex. Obtained results indicate that MI posttreatment after KA induced SE could successfully target the biochemical processes involved in apoptosis, reduces cell loss, and can be successfully used in the future for translational research.

## 1. Introduction

Epilepsy is a heterogeneous syndrome characterized by recurrent and spontaneous seizures. Approximately 1% of the population in the world suffers from epilepsy. However, 20%–30% of the patients are refractory to therapies using currently available antiepileptic drugs (AEDs) [1]. Current epilepsy therapy is symptomatic using AEDs. This therapy suppresses seizures but does not prevent or cure epilepsy. Thus, treatment strategies that could interfere with the process leading to epilepsy (epileptogenesis) would have significant benefits over the current approach [1–3] and will be of great importance for epilepsy treatment. Unfortunately, at present, there is no drug which could fulfill these demands and effectively prevent the process of epileptogenesis in humans. The alternative goal for epileptogenesis treatment would be disease modification, which means that although a treatment may not prevent the occurrence of a disease, it may nevertheless modify the natural course of the disease [1]. Disease modification after epileptogenic brain insults may affect the development of spontaneous seizures in that the seizures, if not prevented, are less frequent and less severe [1].

Some native plants of the Ranunculaceae family (to which plant* Aquilegia vulgaris* belongs) are widely used in Chinese and Tibetan folk medicine as antiepileptic and soporific medicaments [4]. In our early studies we discovered that water extract of* Aquilegia vulgaris* contains compounds which act on *γ*-aminobutyric acid- (GABA-) A receptors; namely, it completely inhibits ^3^H-muscimol (a GABA-A receptor agonist) binding to rat brain membranes and also increases ^3^H-flunitrazepam (a specific ligand for the GABA-A receptor benzodiazepine site) binding by approximately a factor of two [4]. *γ*-Aminobutyric acid (GABA) is the main inhibitory neurotransmitter of the mammalian central nervous system (CNS) and several antiseizure medicines act through the GABA system [5]. GABA itself is unable to penetrate the blood-brain barrier and systemic intraperitoneal administration of GABA is not accompanied by anticonvulsive activity [6]. Therefore it was hypothesized that water extract from* Aquilegia vulgaris* contains active components other than GABA itself. In the next series of our experiments compounds were identified, which inhibit ^3^H-muscimol binding to rat brain membranes and increase ^3^H-flunitrazepam binding in* in vitro* system. High performance liquid chromatography and subsequent gas chromatography coupled with mass spectrometry identified two main active compounds of this extract: (1) myoinositol (MI) and (2) oleamide—sleep inducing lipid [4]. MI was not expected to influence ^3^H-muscimol binding, but we experimentally confirmed that MI is the compound of the fraction inhibiting ^3^H-muscimol binding [4].

In later studies we revealed that MI pretreatment significantly decreases the severity of seizures induced either by pentylenetetrazole (PTZ) or by kainic acid (KA) [7, 8]. In the next series of experiments initially we induced the status epilepticus (SE) by KA and then tried MI daily treatment. Using such design of experiment antiepileptogenic properties of compound could be explored (see for review [1]). We found out that MI treatment during 28 days attenuates biochemical changes underlying the process of epileptogenesis. Namely, KA induced epileptogenesis is accompanied by a strong decrease in the amount of GLUR1 subunit of AMPA-glutamate receptors and calcium-calmodulin dependent protein kinase II (CaMKII) in the hippocampus, which are nearly completely reversed by daily treatment of MI [9]. Our recent data indicated that MI treatment, utilizing the same design of experiment, restores the normal level of gamma-2 subunit of GABA-receptors' amount (mainly found in synapses and participating in phasic inhibitions), which is drastically reduced on the 28th day after KA treatment [10]. MI treatment demonstrated no specific effect on expression levels of GLUR1, CaMKII, or GABA-A receptor subunits 28–30 h after KA induced SE [9, 10]. Nevertheless it is highly plausible that some other biochemical processes are affected by MI treatment at early stages of KA induced SE and epileptogenesis. In the present series of experiments we focused on mitochondrial proteins, enzymatic marker of apoptosis, and also evaluated the cell loss in hippocampus after KA induced SE and MI and diazepam treatment.

The rationale of these experiments was as follows: it is well documented that KA increases mitochondrial dysfunction and apoptosis in neurons of hippocampus (reviewed in [11]). We have shown that MI pretreatment before KA induced SE exerts strong neuroprotective effect on hippocampal cell loss during the process of epileptogenesis and preserves the structure of neurons, synapses, and mitochondria on the 14th day after treatment [12]. We were interested in exploring whether MI treatment after KA induced SE influenced the processes of apoptosis, mitochondrial markers, and cell loss at early stages, namely, on the 1st day after KA administration.

We have addressed these questions by studying (i) changes in the amount of the following proteins: voltage-dependent anionic channel- (VDAC-) 1, VDAC-2, I subunit of cytochrome oxidase c (CO-I), and cofilin; (ii) alterations of the caspase-3 activity; and (iii) hippocampal cell loss.

The above-mentioned proteins were selected for the following reasons. (i) Both VDAC-1 and VDAC-2 are considered as a major regulator of cell death (e.g., for recent reviews see [13–15]). (ii) CO-I is one of the catalytic subunits of the cytochrome c oxidase enzyme complex and is expressed in mitochondria. This enzyme is central to energy metabolism [16], and mitochondria play a role in many aspects of neuronal function and neuropathologies including epilepsy [17–19]. (iii) Cofilin is one of the proteins upregulated during the neuronal death [20, 21] and mitochondrial translocation of cofilin is an early step in apoptosis induction [22]. (iv) Caspase-3 is the main downstream effector caspase that cleaves the majority of the cellular substrates in apoptotic cells [23].

MI significantly reduces the seizure score and seizure duration of KA and PTZ induced convulsions [7, 8] and also inhibits ^3^H-muscimol binding to GABA-A receptors* in vitro* [4]. Thus it is possible that MI effects on biochemical processes of epileptogenesis, observed in our previous experiments [9, 10], were mediated by its action on GABA-A receptors and/or anticonvulsant properties of MI, other than its action on GABA-A receptors. To evaluate this possibility in our present experiments we included a group of animals treated with anticonvulsant and GABA-A receptor agonist diazepam.

## 2. Materials and Methods

### 2.1. KA Induced SE

Male Wistar rats of 2.5–3 months of age received a single intraperitoneal injection of KA (10 mg/kg, Sigma) dissolved in saline. After injection each animal was placed into an individual plastic cage for observation for 4 hours. Seizures were scored according to a modified Racine scoring system from 0 to 6 [9, 10, 24, 25].

Thirty-two animals with seizures of grades 4-5 were selected. Those animals exhibited seizures at least for 60 minutes—which is enough to induce epilepsy for this type of treatment [1]. Six KA treated animals died within the first day and experiments were continued on 26 animals; 8 rats were used for morphological studies and 18 for biochemical experiments.

Twenty-two control rats received saline injections and after that were treated in the same way as KA treated animals; 18 animals for biochemical experiments and 4 animals for morphological studies were selected. To summarize 36 animals were used in biochemical experiments and 12 for morphological studies.

### 2.2. Biochemical Experiments

Eighteen rats treated with KA and 18 rats treated with saline (see above) were divided into six group. Four hours following KA treatment and SE development the 18 animals were divided into three groups 6 in each: the first group received a saline (0.9% NaCl sterile solution) injection (1 mL/kg, KA + SAL group), the second group MI (30 mg/kg) injection (KA + MI group), and the third group diazepam (5 mg/kg) injection (KA + DIAZ group). Each of these groups received the same injections at the end of the first day and in the morning of the following day. The total amount of injections was 3. 6 h later after the last injection the rats were decapitated (time corresponding to the 28–30 h following KA treatment).

**Figure 1:**
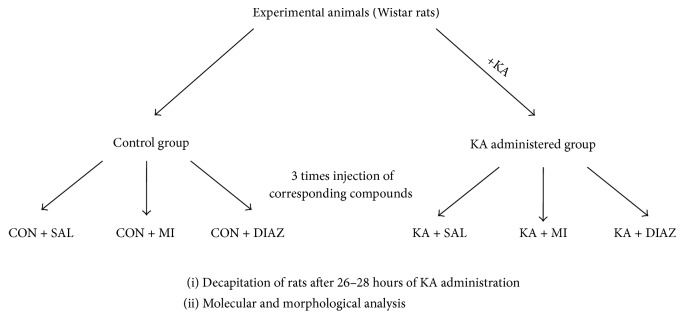
The diagram of experiment design.

Eighteen control animals after saline injections were divided also into three groups 6 in each: the first group received a saline injection (1 mL/kg, CON + SAL group), the second group MI (30 mg/kg) injection (CON + MI group), and the third group diazepam (5 mg/kg) injection (CON + DIAZ group). The time schedule of the remaining treatment and decapitation was exactly the same as for KA treated animals (see above). The diagram of experimental design is provided in Figure 1. During the whole experiment, the rats were housed in cages with free access to water and food. All experiments were carried out at the I. Beritashvili Center of Experimental Biomedicine and were performed in compliance with approved institutional animal care guidelines.

After decapitation, two regions—hippocampus and neocortex—were removed from each brain and immediately frozen in dry ice. The number of animals used was estimated to be the minimum required for adequate statistical analysis [9, 10]. All biochemical experiments were carried out in “blind” manner; samples were then coded and all subsequent procedures were performed without knowledge of the rats' experimental history.

### 2.3. Electrophoresis and Immunoblotting

The tissue samples were rapidly homogenized in 20 mM Tris-HCl (pH 7.4), 0.32 M sucrose, 1 mM ethylenediaminetetraacetic acid, 1 mM sodium orthovanadate, 10 mM sodium pyrophosphate, 0.5 mM ethylene glycol-bis(2-aminoethylether)-N,N,N′,N′-tetraacetic acid, and a cocktail of protease inhibitors (Sigma P8340). One-fourth of the whole homogenate was saved for the determination of caspase-3 activity and the three-quarters (remaining part) were centrifuged at 1000 ×g for 10 min. The supernatant was further centrifuged at 15,000 ×g for 20 min. The pellet was washed once and is referred to as the P2 mitochondrial-membrane fraction. This fraction was dissolved in 5% sodium dodecyl sulfate (SDS) solution.

In all fractions, the protein concentration was determined in quadruplicate using a micro bicinchoninic acid protein assay kit (Pierce). Aliquots containing 30 *μ*g of protein and of equal volume were applied to the gels. SDS gel electrophoresis and Western blotting were carried out as described previously [9, 10]. After the protein had been transferred onto nitrocellulose membranes, the membranes were stained with Ponceau S solution to confirm the transfer and the uniform loading of the gels.

For the detection and quantification of selected proteins the following antibodies were used: (i) for VDAC-1 (Abcam, ab15895); (ii) for VDAC-2 (Abcam, ab37985); (iii) for cofilin (Abcam, ab 42824); (iv) and for CO-I (CO-I; Molecular Probes, A6403). As cofilin was measured in the P2 mitochondrial-membrane fraction it is referred to as M-cofilin.

Due to the close proximity of molecular weights of the VDAC-1 and VDAC-2 separate electrophoresis/Western immunoblottings were carried out for quantification of these proteins. Standard immunochemical procedures were carried out using peroxidase-labeled secondary antibodies and Super-Signal West Pico Chemiluminescent substrate (Pierce) [9, 10]. The optical densities of bands corresponding to the VDAC-1, VDAC-2, M-cofilin, and CO-I were measured using LabWorks 4.0 (UVP). The autoradiographs were calibrated by including in each gel four standards of P2 mitochondrial-membrane fraction from the brain of untreated rats. Each standard contained 15–60 *μ*g of total protein. Optical density was proportional to the amounts of VDAC-1, VDAC-2, M-cofilin, and CO-I (see Figure 3). To obtain the data given in Figures 4 and 5 the optical density of each band from the experimental sample was divided by the optical density which, from the calibration of the same autoradiograph, corresponded to 30 *μ*g of total protein in the standard [9, 10]. Data expressed in this way will be referred to as “relative amount” of protein.

Data from experimental stained protein bands were not normalized with respect to actin or any other housekeeping protein because it cannot be guaranteed that such proteins are unaffected by KA or other treatment [26–28] (for discussion of the unreliability of normalization to housekeeping proteins see [29]). Instead, we controlled loading by Ponceau S staining and calibrated all gels with the same protein standards (see above and also [30]).

### 2.4. Caspase-3 Activity

Caspase-3 enzymatic activity was assayed using a colorimetric caspase-3 assay kit (Sigma-Aldrich, Cat. CASP-3-C) according to the manufacturer's instructions. This kit measures the activity of caspase-3, one of the critical enzymes of apoptosis, and includes a specific inhibitor for precise measurement of caspase-3 activity. The enzyme activity was measured in the total homogenate fractions of rat hippocampus and neocortex separately.

Each tissue sample was assayed in three parallel measurements without caspase-3 inhibitor and in three measurements with inhibitor. Plate wells contained precise amounts of homogenate proteins in the range of 10–12 micrograms and incubated for 2 h at 37°C with caspase-3 peptide substrate, with or without enzyme inhibitor. The amount of* p*-nitroaniline (pNA) released in the assay was measured using a spectrophotometer (OD 405 nm) and the concentration was determined by the standard curve. These values were subtracted from the values obtained without the inhibitor. The enzyme activity is expressed as nanomoles of pNA released per minute per 1 mg of tissue homogenate protein.

### 2.5. Cell Count

Three groups of rats were used to perform cell count: CON + SAL, KA + SAL, and KA + MI. Each group consisted of 4 rats. Animals were deeply anesthetized with ketamine (100 mg/kg) and then perfused transcardially with 4% paraformaldehyde in 0.1 M phosphate buffer (pH 7.4). Excised brains were postfixed at 4°C in the same fixative for another 24 h and then cryoprotected in a 30% sucrose-solution. For Nissl staining 15 *μ*m coronal sections were cut on a cryostat (Microm HM 500 M). Every 6th section was collected and mounted on a poly-L-lysine coated glass slides. The slides were left to dry and rehydrated with 100% alcohol, 95% alcohol, and distilled water. Subsequently, the sections were stained in 0.1% Cresyl violet (Sigma-Aldrich, Cat. number C504) solution. The sections were then differentiated into 95% ethyl alcohol, dehydrated in 100% alcohol, and rinsed in xylene. Finally, the sections were mounted and observed under a light microscope (Leica DM LB). Cell counting in hippocampal CA1, CA3, and dentate gyrus fields was conducted blindly. For this purpose the systematic random sampling was employed. The 2-dimensional counting grid (250 *μ*m × 250 *μ*m) at the magnification 400x was used. Totally 10–12 sections from each level within experimental and control animals were selected (35 randomly chosen ranges of visions at the same site of all sections from each animal). 

### 2.6. Statistical Analysis

#### 2.6.1. Seizure Grades and Seizure Duration Comparison

The seizure grades between three different groups of KA treated rats used in biochemical experiments and two groups of rats used in morphological studies were compared with Mann-Whitney test. The same groups of rats were compared by total duration of seizures; in this case Student's-*t* test was used.

#### 2.6.2. Changes in Protein Amounts and Caspase-3 Activity

Data for each protein and caspase-3 activity were analyzed separately by two-way ANOVA with the following factors: experimental condition (CON + SAL, CON + MI, and CON + DIAZ; KA + SAL, KA + MI, and KA + DIAZ) and brain region (hippocampus and neocortex). Planned comparisons were made between all groups (CON + SAL, CON + MI, and CON + DIAZ; KA + SAL, KA + MI, and KA + DIAZ) at a defined region (hippocampus or neocortex).

#### 2.6.3. Cell Count

Data for cell counts was analyzed by two-way ANOVA with the following factors of experimental condition (CON + SAL, KA + SAL, and KA + MI) and hippocampus subfield (CA1, CA3, and dentate gyrus). Planned comparisons were made between these groups at a defined hippocampus subfield (e.g., CA1).

All statistical tests were two-tailed and all significant differences are reported.

## 3. Results

### 3.1. Seizure Grade and Duration

The seizure grades in three groups of KA treated rats used in biochemical experiments were compared with each other and no significant differences were found between them (Mann-Whitney test—KA + SAL versus KA + MI, *W* = 39.0, *p* = 1.0; KA + SAL versus KA + DIAZ, *W* = 42.0, *p* = 0.64; and KA + MI versus KA + DIAZ, *W* = 42.0, *p* = 0.64). The medians for KA + SAL and KA + MI groups were 5.0 and for KA + DIAZ group 4.5.

The two groups of rats used in morphological studies consisted of 4 animals and by the seizure grade they were not significantly different from each other (Mann-Whitney test—KA + SAL versus KA + MI, *W* = 16.0, *p* = 0.61). The median for KA + Sal was 4.5 and for KA + MI 5.0.

**Figure 2:**
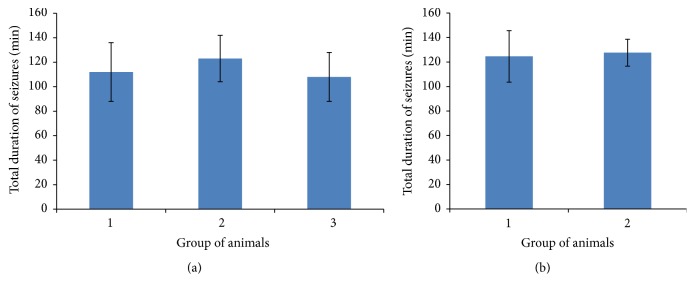
Total duration of seizures (min) in various group of animals. The duration of observable seizure activity was measured for each animal and mean values ± standard error of the mean are provided for each group. (a) Groups of animals used in biochemical experiments; (b) groups of animals used in morphological studies. 1: KA + SAL; 2: KA + MI; and 3: KA + DIAZ. Groups were compared using a two-tailed *t*-test. No significant differences were found (*p* > 0.6 for each comparison).

The groups of rats for biochemical and morphological experiments were not significantly different from each other by total duration of seizures (see Figure 2).

Approximately 4.5 h after KA treatment all groups of rats were video-monitored by infrared cameras till decapitation. No recurrent seizures were observed in any group of rats.

### 3.2. Immunostaining

**Figure 3:**
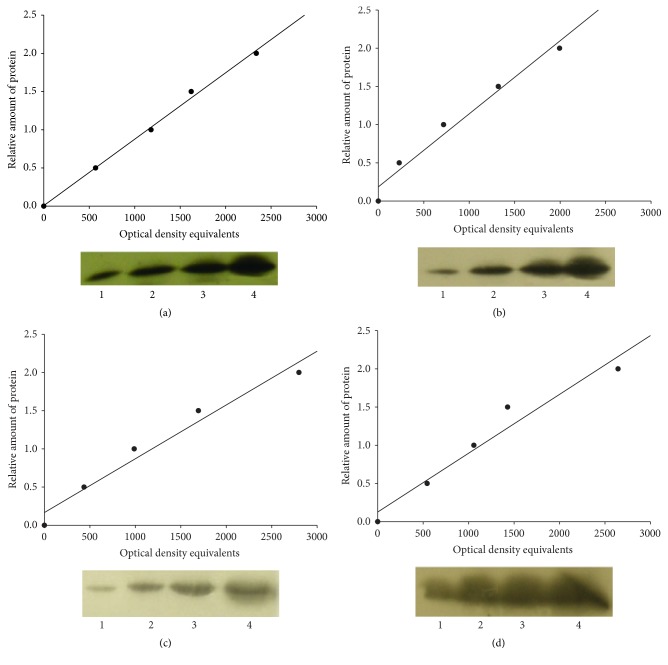
Sample films and calibration plots for (a) VDAC-1; (b) VDAC-2; (c) CO-I; and (d) M-cofilin. Bottom panels: sample radiographs; top panels: calibration plots (lines fitted by linear least-squares regression).

Anti-VDAC-1 antibodies bound to a band of molecular weight 30 kDa, anti-VDAC-2 stained a protein band with molecular weight 31 kDa, anti-CO-I antibodies reacted with a protein band of molecular weight 57 kDa, and anti-cofilin antibodies stained a protein band of molecular weight 17 kDa (Figure 3). All of these weights corresponded to the expected size of the target proteins.

Four standards (15, 30, 45, and 60 *μ*g of total protein) were applied to each gel. For these standards the optical densities of the immunostained bands (for VDAC-1, VDAC-2, CO-I, or M-cofilin) were plotted against the amounts of protein; in all these standards, least-squares regression showed a significant fit to a straight line (Figure 3).

Thus the used antibodies are specifically reacting against target proteins and measured optical densities are quantitatively reflecting the amounts of proteins.

#### 3.2.1. VDAC-1

**Figure 4:**
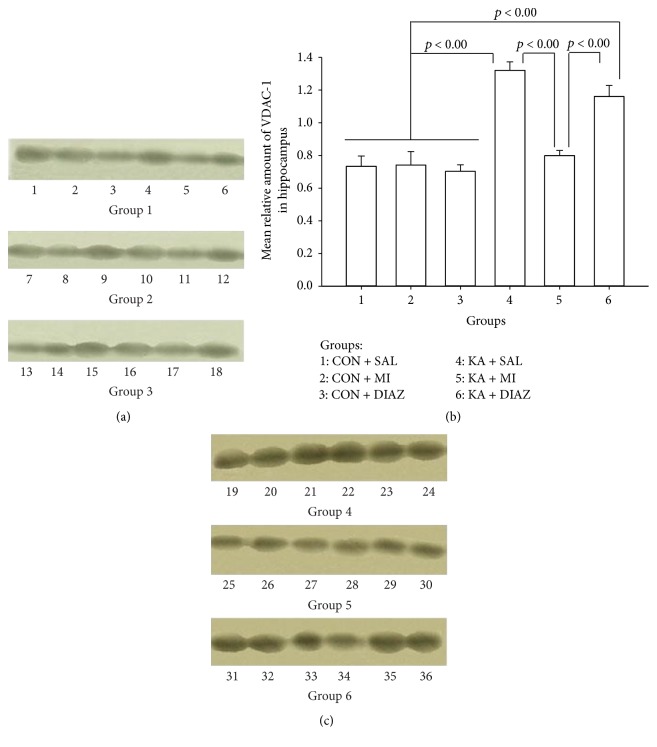
Representative Western blot autoradiograph of P2 mitochondrial-membrane fraction for VDAC-1, (a) and (c), and mean relative amounts of VDAC-1 (b) from the hippocampus of 6 different groups of rats. (a) and (c) Each lane was derived from a single sample. Lanes 1–6: CON + SAL; lanes 7–12: CON + MI; lanes 13–18: CON + DIAZ; lanes 19–24: KA + SAL; lanes 25–30: KA + MI; and lanes 31–36: KA + DIAZ. (b) Error bars represent the standard errors of the means. KA + SAL and KA + DIAZ groups significantly exceed all control groups as well as KA + MI group (*p* < 0.000). The mean amount of VDAC-1 in the KA + MI group is not significantly different from the means of any of the control groups and the means of control groups differ significantly from each other. Groups of rats: 1: CON + SAL; 2: CON + MI; 3: CON + DIAZ; 4: KA + SAL; 5: KA + MI; and 6: KA + DIAZ.

Two-way ANOVA revealed a significant effect of experimental condition on the amount of VDAC-1 (*F*_5,71_ = 5.42, *p* < 0.0001). The effect of the factor-region was not significant, but the interaction between these two factors was significant (*F*_5,71_ = 7.75, *p* < 0.0001). The significant differences between the different groups of animals were found only in the hippocampus. The mean amount of VDAC-1 in KA + SAL group was significantly higher as compared to all control groups (KA + SAL versus CON + SAL, *t* = 7.24, *p* < 0.0001; KA + SAL versus CON + MI, *t* = 5.95, *p* < 0.0001; and KA + SAL versus CON + DIAZ, *t* = 9.40, *p* < 0.0001; for all comparisons df = 10) and also significantly higher as compared to KA + MI group (*t* = 8.47, *p* < 0.0001, and df = 10). The KA + DIAZ group was indistinguishable from KA + SAL group and the mean amount of VDAC-1 significantly exceeded the mean amounts of the control groups (KA + DIAZ versus CON + SAL, *t* = 4.59, *p* = 0.001; KA + DIAZ versus CON + MI, *t* = 3.90, *p* = 0.003; and KA + DIAZ versus CON + DIAZ, *t* = 5.71, *p* < 0.0001; for all comparisons df = 10) and also was significantly higher as compared to KA + MI group (*t* = 4.71, *p* = 0.001, and df = 10). The mean value of VDAC-1 in KA + MI group was not different from the corresponding values in any control group. Thus KA induced increase in VDAC-1 amount is abolished by MI posttreatment but is not changed by diazepam treatment (see Figure 4).

In neocortex no significant differences were found.

#### 3.2.2. VDAC-2

No significant changes were revealed in ANOVA and no differences were significant between the groups.

#### 3.2.3. CO-I

There was not any significant effect of factors experimental conditions or region in ANOVA on the amounts of CO-I and no differences were significant between the groups.

#### 3.2.4. M-Cofilin

**Figure 5:**
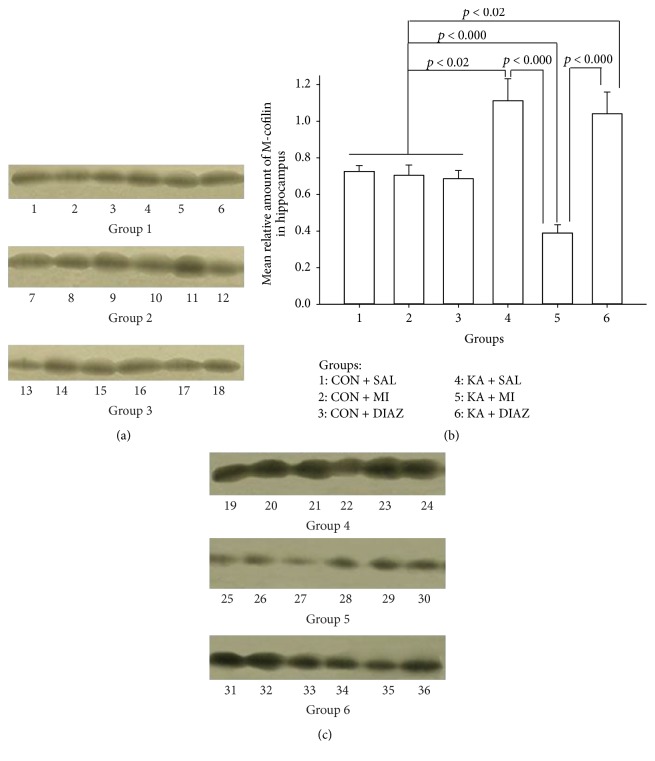
Representative Western blot autoradiograph of P2 mitochondrial-membrane fraction for M-cofilin, (a) and (c), and mean relative amount of M-cofilin (b) from the hippocampus of 6 different groups of rats. (a) and (c) Each lane was derived from a single sample. Lanes 1–6: CON + SAL; lanes 7–12: CON + MI; lanes 13–18: CON + DIAZ; lanes 19–24: KA + SAL; lanes 25–30: KA + MI; and lanes 31–36: KA + DIAZ. (b) Error bars represent the standard errors of the means. KA + SAL and KA + DIAZ groups significantly exceed all control groups (*p* < 0.002) as well as KA + MI group (*p* < 0.000). The mean amount of M-cofilin in the KA + MI group is significantly lower as compared to the means of any of the control groups (*p* < 0.000). The means of control groups do differ significantly from each other. Groups of rats: 1: CON + SAL; 2: CON + MI; 3: CON + DIAZ; 4: KA + SAL; 5: KA + MI; and 6: KA + DIAZ.

For the amount of M-cofilin the effects of both factors (experimental condition and region) were significant (*F*_5,71_ = 4.84, *p* = 0.001, and *F*_1,71_ = 25.06, *p* < 0.0001, resp.) and interaction between these factors was also significant (*F*_5,71_ = 4.80, *p* = 0.001). These effects in ANOVA were due to the changes in hippocampus. The mean amount of M-cofilin in the KA + SAL treated groups of hippocampus was significantly higher as compared to all other groups except the KA + DIAZ group (KA + SAL versus CON + SAL, *t* = 3.02, *p* = 0.013; KA + SAL versus CON + MI, *t* = 3.00, *p* = 0.013; KA + SAL versus CON + DIAZ, *t* = 3.24, *p* = 0.009; and KA + SAL versus KA + MI, *t* = 5.49, *p* < 0.0001; for all comparisons df = 10). The changes in KA + DIAZ group were essentially the same as in KA + SAL group and the mean amount of M-cofilin in KA + DIAZ group significantly exceeded the mean amounts of other groups (KA + DIAZ versus CON + SAL, *t* = 2.63, *p* = 0.025; KA + DIAZ versus CON + MI, *t* = 2.62, *p* = 0.026; KA + DIAZ versus CON + DIAZ, *t* = 2.86, *p* = 0.017; and KA + DIAZ versus KA + MI, *t* = 5.25, *p* < 0.0001; for all comparisons df = 10). Treatment of KA group with MI not only prevents the increase of the M-cofilin (see comparisons above) but also significantly decreases the level of protein as compared to all control groups (KA + MI versus CON + SAL, *t* = 6.08, *p* < 0.0001; KA + MI versus CON + MI, *t* = 4.36, *p* = 0.001; and KA + MI versus CON + DIAZ, *t* = 4.69, *p* = 0.001; for all comparisons df = 10) (Figure 5).

No differences were significant in neocortex.

### 3.3. Caspase-3

**Figure 6:**
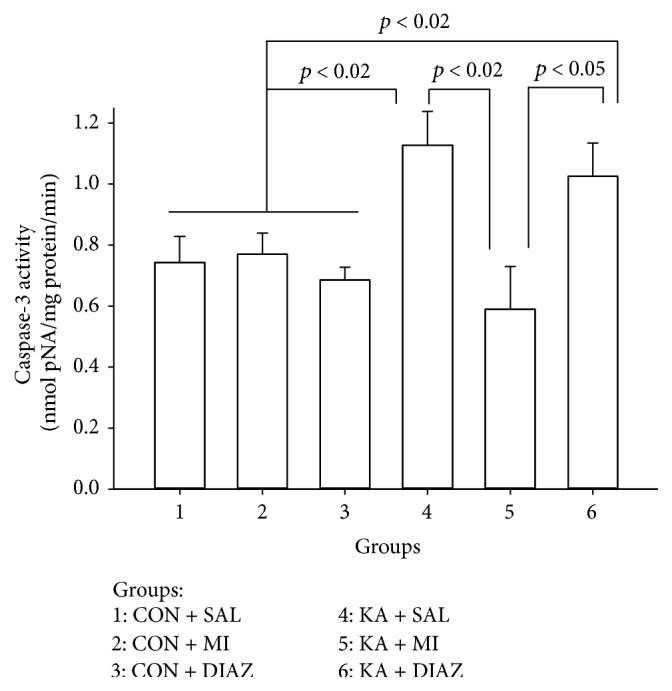
The mean amount of caspase-3 activities in the hippocampus of different experimental groups of rats. Error bars represent the standard errors of the means. KA + SAL and KA + DIAZ groups significantly exceed all control groups (*p* < 0.002) as well as KA + MI group (*p* < 0.002 and *p* < 0.005, resp.). The mean amount of enzyme activity in the KA + MI group does not significantly differ from the means of any of the control groups and the mean of control groups does significantly differ from each other. Groups of rats: 1: CON + SAL; 2: CON + MI; 3: CON + DIAZ; 4: KA + SAL; 5: KA + MI; and 6: KA + DIAZ.

Two-way ANOVA revealed a significant effect of experimental condition and region on the changes of caspase-3 activity (*F*_5,71_ = 3.55, *p* = 0.007, and *F*_1,71_ = 9.53, *p* = 0.003, resp.). The interaction between these two factors was also significant (*F*_5,71_ = 4.79, *p* = 0.001). The changes in caspase-3 activity are analogous to the changes of the amount of VDAC-1 and M-cofilin. The highest activity of the enzyme is observed in the hippocampus of KA + SAL and KA + DIAZ groups. KA + SAL group significantly exceeds all control as well as KA + MI groups (KA + SAL versus CON + SAL, *t* = 2.72, *p* = 0.021; KA + SAL versus CON + MI, *t* = 2.73, *p* = 0.021; KA + SAL versus CON + DIAZ, *t* = 3.70, *p* = 0.004; and KA + SAL versus KA + MI, *t* = 3.03, *p* = 0.013; for all comparisons df = 10) (Figure 6).

The mean amount of caspase-3 activity in KA + DIAZ is significantly higher as compared to CON + DIAZ group (*t* = 2.93, *p* = 0.015, and df = 10) and as compared to KA + MI group (*t* = 2.49, *p* = 0.032, and df = 10), whereas the differences with CON + SAL and CON + MI groups are significant only on one-tailed *t*-test (*t* = 2.04, *p* = 0.035, and *t* = 2.00, *p* = 0.037, resp.; for both cases df = 10) (Figure 6).

No differences were significant between neocortex samples.

### 3.4. Cell Number in Hippocampal Subfields

There was a strong effect of experimental condition and subfield on the number of neurons in hippocampus in two-way ANOVA (training condition factor *F*_2,35_ = 142.76, *p* < 0.0001; subfield factor *F*_2,35_ = 67.84, *p* < 0.0001). The interaction between these two factors was also significant (*F*_4,35_ = 34.28, *p* < 0.0001). The planned comparison between the groups of subfields was carried out separately.

#### 3.4.1. CA1

**Figure 7:**
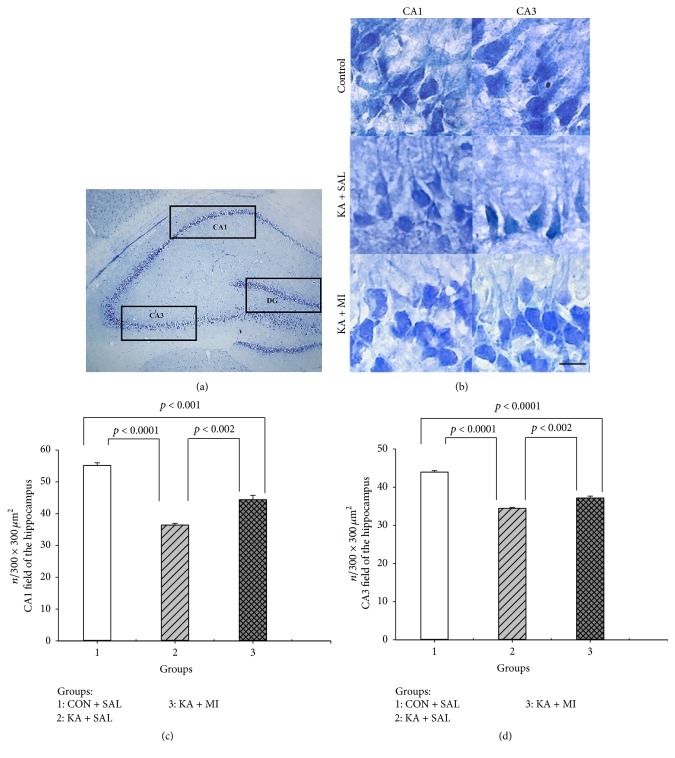
The view of hippocampus subfields, where cell count was performed (a). Hippocampal CA1 (left) and CA3 (right) subfields of CON + SAL, KA + SAL, and KA + MI treated animals (b). Mean numbers of neurons in the CA1 (c) and CA3 (d) subfields of the hippocampus in three different groups of animals. (b) The photomicrographs demonstrate the obvious decrease of neuronal cells in CA1 (a) and CA3 (b) subfields of hippocampus as a result of KA + SAL treatment and partial rescue of the cells in KA + MI group. (c) and (d) Mean number of neurons (number of cell counts per counting frame area (250 × 250 *μ*m^2^)) in the CA1 and CA3 subfields of the hippocampus. Error bars represent the standard errors of the means. In the CA1 subfield the mean number of cells in KA + SAL groups is significantly less as compared to CON + SAL group (*p* < 0.000) as well as compared to KA + MI group (*p* = 0.001). The CON + SAL group also significantly exceeds the KA + MI group (*p* = 0.002). In the CA3 subfield of hippocampus the mean number of cells in CON + SAL is significantly higher as compared to KA + SAL as well as compared to KA + MI group (for both comparisons *p* < 0.0001). KA + MI group significantly exceeds the KA + SAL group (*p* = 0.002). Scale bar = 15.

The KA treatment significantly reduces the number of neurons by more than 30% as compared to control rats (*t* = 17.90, *p* = 0.000, and df = 6). In the KA + MI group the reduction of cell number is less (<20%) but still significantly different from control group (*t* = 6.58, *p* = 0.001, and df = 6). The difference between the KA + MI and KA + SAL groups is also significant (*t* = 5.43, *p* = 0.002, and df = 6). Thus MI treatment significantly reduces the KA induced neuronal cell loss in the CA1 subfield of hippocampus (Figure 7).

#### 3.4.2. CA3

The significant decrease in neuronal cell number after KA induced SE as compared to control group is also observed in CA3 subfield (>20%; *t* = 21.41, *p* < 0.0001, and df = 6). In KA + MI group the decrease is also significant (*t* = 11.32, *p* < 0.0001, and df = 6), but less (15%). The difference between the KA + SAL and KA + MI group is significant (*t* = 5.33, *p* = 0.002, and df = 6), indicating that MI also exerts neuroprotective effect in CA3 subfield of hippocampus (Figure 7).

#### 3.4.3. Dentate Gyrus

There are no significant differences between the groups; however the rank order of the means is the same as for CA1 and CA3 subfields: CON + SAL > KA + MI > KA + SAL.

## 4. Discussion

In our earlier studies we demonstrated that long-term (28 days), daily treatment of rats with MI after KA induced SE prevents some biochemical processes of epileptogenesis [9, 10]. These biochemical processes included changes in different subunits of GABA-A receptors, GLUR1 subunit of AMPA-glutamate receptors and *α*-subunit of CaMKII [9, 10]. However MI had no influence on these biochemical processes one day after SE [9, 10] suggesting that some other molecular processes are affected at early phases of MI treatment. In our previous experiments MI was applied before KA induced SE and neuroprotective properties of MI on hippocampal cell loss and structure of neurons were elucidated [12]. The major goal of our research is to find the ways to prevent processes of epileptogenesis or to modify it since the process is already initiated. Therefore the main interest of present studies was to investigate MI effects after KA induced SE, especially at its early phases (1 day). Based on our previous data [12] we have hypothesized that MI could prevent the neuronal loss and processes of apoptosis even when the treatment starts after SE and obtained data supports this suggestion.

The spontaneous recurrent seizures after KA induced SE start after 7–10 days [31]. Therefore with our experimental design (1 day after SE) it was impossible to detect which experimental animals would develop recurrent seizures. However, in our experiments, we included only those rats which displayed seizures at least for 60 min during first 4 h after KA treatment that should be enough for the development of epilepsy [1]. Indeed our unpublished observation indicates that more than 90% of such rats develop recurrent seizures.

Do the observed changes reflect biochemical processes of epileptogenesis? One of the major challenges to the epilepsy research community indeed has been to determine which of the molecular changes after SE contribute to epileptogenesis, which are compensatory, and which are noncontributory [32]. Our results have mainly revealed molecular changes associated with apoptosis and cell loss. Could be these changes unequivocally associated with the processes of epileptogenesis? The effect of seizures on neuronal death and the role of seizure-induced neuronal death in acquired epileptogenesis have been debated for decades (e.g., for reviews see [33–35]). According to the hypothesis of “*recapitulation of development*” a loss of synaptic input from the dying neurons is a critical signal to induce axonal sprouting and synaptic-circuit reorganization [33]. According to the* “neuronal death pathway” hypothesis*, the biochemical pathways causing programmed neurodegeneration, rather than neuronal death* per se*, are responsible for or contribute to epileptogenesis [33]. Thus we could only suggest that reported molecular changes and the effects of MI could be linked to the epileptogenesis.

### 4.1. VDACs

VDACs are the most abundant proteins of mitochondrial outer membrane through which a continuous bidirectional transport of ATP, ADP, NADH, and ions and general metabolite flux take place. Mitochondrial outer membrane is responsible for maintaining mitochondrial integrity, which may otherwise lead to cellular dysfunctions, including cessation of ATP synthesis, dysregulation of Ca^2+^ homeostasis, release of cytochrome c, and apoptosis [36, 37]. There are three isoforms of VDAC in eutherian mammals. VDAC-1 is characterized by the highest expression levels in most cell types and is considered to possess proapoptotic properties [36, 37], whilst VDAC-2 and VDAC-3 are considered to be positive regulators of ferroptosis, but not of apoptosis [15].

The obtained data point out to significant changes in VDAC-1, but not in VDAC-2 after KA induced SE in the hippocampus of rats. The mean amount of VDAC-1 is increased in KA + SAL and KA + DIAZ groups as compared to all control and also to KA + MI groups. The latter group does not differ from control groups. Thus, MI treatment 4 h later after KA injection abolishes increase of VDAC-1 in the hippocampus. We suggest that increase of VDAC-1 in KA + SAL and KA + DIAZ groups should be the indicator of increased apoptosis, and MI treatment prevents this process.

### 4.2. CO-I

We have not found any significant changes in the expression of CO-I. The cytochrome c oxidase is the marker of the mitochondrial complex IV and the CO-I is encoded by mitochondrial DNA. It was also shown that KA induced SE within 3 hours of KA administration produces decrease in the activity of respiratory complex I, but not of complex IV [38].

The increase in VDAC-1, but not changes in CO-I and VDAC-2, indicates that changes in VDAC-1 are specific for mitochondria after KA induced SE. Thus mitochondria are undergoing specific changes after KA treatment.

### 4.3. M-Cofilin

Cofilin is a well-known regulator of actin filament nonequilibrium assembly and disassembly (e.g., for review see [39]). Dephosphorylation of cofilin is linked to subsequent increase of cofilin-actin binding and actin depolymerization. This is one of the cellular mechanisms leading to dendritic spine loss in the pilocarpine model of SE [40]. The second function of cofilin is not related to the regulation of actin assembly and involves translocation to the mitochondria and induction of apoptosis [22, 39]. In the present study we studied the changes in the amount of M-cofilin in the mitochondria-plasma membrane—P2 fraction. The selection of this fraction for present experiments had another reason too—in our previous experiments we compared hippocampal P2 fraction protein extracts of KA + SAL and KA + MI groups of rats using 2-dimensional electrophoresis. One of the proteins which was drastically upregulated in KA + SAL group as compared to KA + MI group was identified by mass spectrometry as cofilin (unpublished data).

Our present data indicate significant increase of M-cofilin in KA + SAL and KA + DIAZ groups as compared to all control groups as well as to KA + MI group. MI treatment after KA induced SE not only prevents the increase in the amount of M-cofilin, but also significantly reduces it as compared to control groups. We suggest that decrease of M-cofilin in P2 mitochondrial-membrane fraction reflects the reduced amount of protein translocated to mitochondria and consequently inhibits the KA induced processes of apoptosis. This suggestion is also supported by another series of our results (see below).

### 4.4. Caspase-3 Activity

Caspase-3 activity is significantly upregulated in the hippocampus of KA + SAL and KA + DIAZ groups as compared to control and KA + MI groups. As in the previous cases (VDAC-1, M-cofilin) MI significantly prevents this increase. Caspase-3 cleaves the majority of cellular substrates in apoptotic cells and is considered to be the main downstream effector caspase. Thus obtained data convincingly demonstrates intensification of apoptosis after KA induced SE in hippocampus and inhibitory effect of MI treatment on it.

What could be the possible causal relationship between VDAC-1, M-cofilin, CO-1, and caspase-3? In our experiments KA treatment induces increase in VDAC-1, M-cofilin, and caspase-3 activity, with no changes in CO-1 amount. Increase in the amount of VDAC-1 and M-cofilin could lead to the changes in mitochondrial permeability transition pore, with increased escape of cytochrome c to cytoplasm and activation of caspase cascade, including caspase-3. It is interesting to note that the same types of changes were observed in rat brain with endurance treadmill training—this exercise decreases the amounts of VDAC-1, cofilin, and caspase-3 alongside with other markers of oxidative stress and apoptotic signaling, whereas no changes were found in CO-I [41].

### 4.5. Cell Number Changes

For VDAC-1, M-cofilin, and caspase-3 activity changes after KA induced SE and MI treatment effects were observed only in the hippocampus. The same is true for CaMKII, GLUR1, and GABA-A receptor subunits' changes in our previous studies [9, 10]. In the present study, according to molecular changes, KA + SAL group did not differ from KA + DIAZ group and therefore cell count studies were carried only in the hippocampus and only in three groups of animals: CON + SAL, KA + SAL, and KA + MI. 

KA induced SE is accompanied by a significant cell number decrease in the CA1 and CA3 subfields of hippocampus as compared to control group. In the KA + MI group for both subfields the neuronal cell number was significantly higher as compared to KA + SAL group, but also significantly less as compared to CON + SAL group. Thus, MI posttreatment after KA induced SE decreases the cell death significantly, though not till the control level. In the DG no significant changes were found; however the rank order of the means was CON + SAL > KA + MI > KA + SAL. It is possible that the same processes take place in DG as in the CA1 and CA3 subfields, but the magnitude of changes is much smaller and our approach could not detect them on a significant level.

MI is characterized by anticonvulsant properties and this inositol could partially reduce the seizure grade and duration of PTZ or KA induced convulsions [7, 8]. The influence of inositol on molecular changes after SE could be simply mediated by antiseizure activity of compound and/or its action on GABA-A receptors. To elucidate this possibility in present experiments we have included additional two groups of animals which were treated by GABA-A receptor agonist and anticonvulsant diazepam. In our experiments we started to administer MI 4 h after KA injection. At this time point behavioural seizures are already absent. The first administration is followed by 2 more injections. Taking into account that behavioural seizures are already finished and administration of MI took place 3 times we decided to use moderate doses of diazepam in control groups. If diazepam treatment had revealed similar effects as MI, we would have assumed that inositol influence was due to its action on GABA-A receptors and/or its anticonvulsant properties. KA + DIAZ group is identical to KA + SAL group and we propose that the influence of MI is mediated by its other mode of action (discussed below).

It should be emphasized that the same intensity of MI administration to the group of rats not treated by KA does not produce any significant changes in the amounts of studied proteins or enzyme activity. Thus, MI mediates specific influence on molecular machinery induced by KA treatment and we consider these changes as the markers of the rescue from apoptosis and neurodegeneration.

In conclusion, our obtained biochemical and morphological data for the first time indicates that MI posttreatment inhibits KA induced cell death apoptosis and related molecular processes in the hippocampus of rats. As well as in our previous studies [9, 10] effects of MI treatment are not observed in neocortex and thus the MI action should be specific for hippocampus.

It is well documented that alteration in MI deposition may play a role in a number of neuropathological conditions including epilepsy, either as a physiologically important osmolyte or as a precursor molecule for phosphoinositide synthesis (reviewed in [42, 43]). Lithium is characterized by proconvulsant effects, which has been attributed to its ability to block the action of inositol monophosphatase, and by concomitant decrease of free inositol. These proconvulsant effects of lithium can be reversed by MI [44, 45].

Soman is an organophosphorus nerve agent that acts as an irreversible inhibitor of acetylcholinesterase. One of the consequences of soman action is convulsive seizures. The strong decrease in the MI levels during 72 h takes place after soman induced seizures. Combined ketamine and atropine treatment shortly after soman injection prevents the decrease in MI level [46].

Experiments on KA induced seizures have shown that Na^+^/MI cotransporter is upregulated in various parts of hippocampus shortly after seizures [47]. In temporal lobe epilepsy patients' seizure focus has an increased level of MI, whereas areas of seizure spread have a lowered level of MI [48]. Gene coding for myoinositol monophosphatase 2 is likely to be febrile seizure susceptibility gene [49]. Thus, changes in MI metabolism are involved in different types of epilepsy and MI system could be important target for successful therapeutic approaches.

We think that MI effects on VDAC-1 and M-cofilin amounts and on caspase-3 activity alterations after KA induced SE could not account for direct action of inositol on the processes of gene expression, protein cleavage, or enzyme inhibition. The general target of MI action could be the normalization of disturbed cellular condition mediated by its osmolyte properties [50, 51]. During intense neuronal excitation (such as seizures) massive influx of Na^+^, Ca^2+^, and Cl^−^ takes place, which leads to water inward flow and cellular swelling [52, 53]. Under such conditions normal enzyme functioning is significantly disturbed [54]. To compensate this disturbed condition neuronal cells accumulate high concentrations of small organic osmolytes, which do not perturb the functioning of the enzymes and could restore the normal cellular conditions [50, 51]. We suggest that for the normalization of hippocampal cell functioning, after KA insult, higher amounts of MI (or some other osmolytes) are required and MI administration in our experiments supplies these required amounts. MI is actively transported via Na^+^/MI cotransporter (see [47]) and at least partially rescues hippocampal cells from apoptosis and other types of cell death.

According to our data MI long-term treatment prevents biochemical changes of epileptogenesis [9, 10]. Present findings point to one of the earlier effects of MI action, namely, the inhibition of apoptosis and attenuation of neuronal cell loss.

Obtained results indicate the early mechanisms of MI action on the processes summoned by KA induced SE and shed a new light on unexpected activities of inositol. The results of the present work could pave the way for the enhancement of neuroprotective and antiapoptotic features of MI and support the potential development of new and effective antiepileptic drugs.
